# Supplementary material for: Association of Maternal Plasma Total Cysteine and Growth among Infants in Nepal: A Cohort Study
Source: Nutrients. 2020 Sep 17;12(9):2849. doi: 10.3390/nu12092849 (PMC7551827; doi:10.3390/nu12092849)
Supplement: Supplementary file 1 [file nutrients-12-02849-s001.pdf]

nutrients

MDPI

Supplementary material

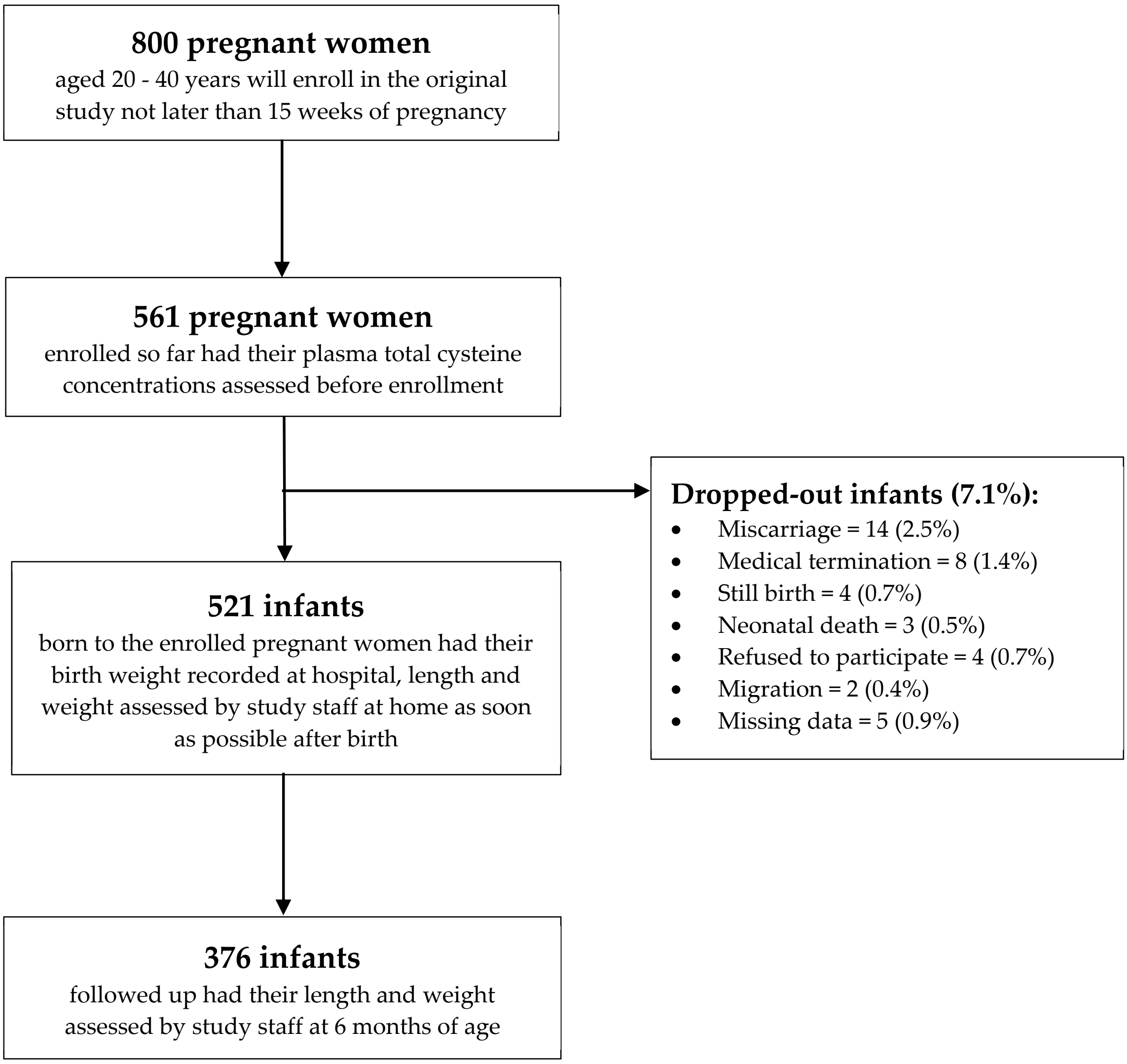

Figure S1. Study participant flow chart.

Table S1. Multivariate linear regression models for anthropometric measurements and maternal plasma total cysteine (tCys) concentration (in µmol/L) categories - low (<25th percentile), reference (25th-75th percentile) and high (>75th percentile).

Anthropometric Indices	n	Crude β-coefficients (95% CI) for tCys		Adjusted β-coefficients (95% CI) for tCys	
		Low	High	Low	High
Birth weight[1], g	521	-7.7 (-98.2, 82.8)	-66.7 (-156.9, 23.5)	-	-
Boys		-	-	26.2 (-69.2, 121.6)	-105.6 (-204.7, -6.4)**
Girls		-	-	-49.4 (-146.7, 47.8)	-11.4 (-111.9, 89.1)
LAZ score at birth	521	0.15 (-0.07, 0.38)	-0.17 (-0.39, 0.06)	0.15 (-0.07, 0.38)	-0.17 (-0.39, 0.06)
WLZ score at birth[2]	503	-0.20 (-0.44, 0.03)*	-0.04 (-0.28, 0.20)	-0.23 (-0.47, 0.01)*	-0.07 (-0.31, 0.17)
LAZ score at 6 months[3]	376	-0.09 (-0.31, 0.14)	-0.18 (-0.41, 0.04)	-0.17 (-0.40, 0.07)	-0.15 (-0.38, 0.07)
WLZ score at 6 months[4]	376	0.002 (-0.26, 0.26)	0.10 (-0.15, 0.36)	.04 (-0.22, 0.30)	0.07 (-0.19, 0.33)

[1] Adjusted for maternal BMI, infant's gender and interaction between tCys and infant's gender. [2.] Adjusted for maternal BMI, education and parity. [3] Adjusted for maternal BMI, parity, WAMI, plasma cobalamin and folate concentrations (*n* = 371 for adjusted tCys because of missing WAMI-index values). [4] Adjusted for maternal BMI, parity and plasma cobalamin concentration. * p-value < 0.10. ** p-value < 0.05. BMI, body mass index; CI, confidence interval; LAZ score, length-for-age Z-score; WLZ score, weight-for-length Z-score.

Table S2. Multivariate linear regression models for anthropometric measurements among different gender and maternal plasma total cysteine (tCys) concentration (in µmol/L).

Anthropometric Indices	n	Crude β-coefficients (95% CI) for tCys	Adjusted β-coefficients (95% CI) for tCys
**Boys**			
Birth weight[1], g	279	-2.294 (-4.283, -0.304)**	-2.567 (-4.565, -0.569)**
LAZ score at birth	279	-0.008 (-0.013, -0.003)***	-0.008 (-0.013, -0.003)***
WLZ score at birth[2]	269	0.001 (-0.005, 0.006)	-0.0001 (-0.005, 0.005)
LAZ score at 6 months[3]	212	-0.005 (-0.010, 0.0003)*	-0.002 (-0.007, 0.003)
WLZ score at 6 months[4]	212	0.002 (-0.004, 0.008)	0.001 (-0.005, 0.007)
**Girls**			
Birth weight[1], g	242	0.822 (-1.435, 3.079)	0.455 (-1.788, 2.698)
LAZ score at birth	242	-0.002 (-0.007, 0.004)	-0.002 (-0.007, 0.004)
WLZ score at birth[2]	234	0.006 (-0.001, 0.012)*	0.006 (-0.0001, 0.013)*
LAZ score at 6 months[3]	164	0.001 (-0.005, 0.007)	0.0004 (-0.006, 0.006)
WLZ score at 6 months[4]	164	0.003 (-0.004, 0.009)	0.001 (-0.005, 0.008)

[1] Adjusted for maternal BMI. [2] Adjusted for maternal BMI, education and parity. [3] Adjusted for maternal BMI, parity, WAMI, plasma cobalamin and folate concentrations (*n* = 209 for boys & 162 for girls for adjusted tCys because of missing WAMI-index values). [4] Adjusted for maternal BMI, parity and plasma cobalamin concentration. * p-value < 0.10. ** p-value < 0.05. *** p-value < 0.01. BMI, body mass index; CI, confidence interval; LAZ score, length-for-age Z-score; WLZ score, weight-for-length Z-score.
